# Performance Assessment for the Validation of Wireless Communication Engines in an Innovative Wearable Monitoring Platform †

**DOI:** 10.3390/s24092782

**Published:** 2024-04-26

**Authors:** Alessio Serrani, Andrea Aliverti

**Affiliations:** 1 Dipartimento di Elettronica, Informazione e Bioingegneria, Politecnico di Milano, 20133 Milano, Italy; 2 Kalpa S.r.l., 20099 Sesto San Giovanni, Italy

**Keywords:** continuous monitoring, wearable platform, custom wireless protocol, BLE protocol, performance assessment, diagnostics, wireless communication, throughput

## Abstract

In today’s health-monitoring applications, there is a growing demand for wireless and wearable acquisition platforms capable of simultaneously gathering multiple bio-signals from multiple body areas. These systems require well-structured software architectures, both to keep different wireless sensing nodes synchronized each other and to flush collected data towards an external gateway. This paper presents a quantitative analysis aimed at validating both the wireless synchronization task (implemented with a custom protocol) and the data transmission task (implemented with the BLE protocol) in a prototype wearable monitoring platform. We evaluated seven frequencies for exchanging synchronization packets (10 Hz, 20 Hz, 30 Hz, 40 Hz, 50 Hz, 60 Hz, 70 Hz) as well as two different BLE configurations (with and without the implementation of a dynamic adaptation of the BLE Connection Interval parameter). Additionally, we tested BLE data transmission performance in five different use case scenarios. As a result, we achieved the optimal performance in the synchronization task (1.18 ticks as median synchronization delay with a Min-Max range of 1.60 ticks and an Interquartile range (IQR) of 0.42 ticks) when exploiting a synchronization frequency of 40 Hz and the dynamic adaptation of the Connection Interval. Moreover, BLE data transmission proved to be significantly more efficient with shorter distances between the communicating nodes, growing worse by 30.5% beyond 8 m. In summary, this study suggests the best-performing network configurations to enhance the synchronization task of the prototype platform under analysis, as well as quantitative details on the best placement of data collectors.

## 1. Introduction

Wearable monitoring platforms (also referred as Body Sensor Networks (BSNs)) are widely used in the healthcare scenario to collect bio-signals and to elaborate physiological data, providing significant help in specific clinical cases. The flexibility of these systems allows their use in a wide range of different diagnostic scenarios. Many different use cases can be found in the literature as well as on the commercial market, most frequently in the cardiovascular field, with a wide variety of systems suitable for electrocardiogram (ECG) and photoplethysmogram (PPG) acquisition [1]. Other common use cases are in the rehabilitation and neurology fields, with platforms designed to collect electromyography (EMG) [2] and electroencephalogram (EEG) [3] signals.

Most of the time, the investigated clinical use case requires the acquisition of multiple bio-signals from different body areas at the same time, meaning with a common time base. In recent years, this requirement has led to the design and development of acquisition platforms based on multi-node architectures, with different sensing nodes specialized for different bio-signals. In order to fulfill the strict requirements around the quality of the collected data in the healthcare context, these kinds of platforms must ensure reliable synchronization among different acquisition channels. Indeed, in a multi-node scenario greater signal synchronization indicates higher data quality on the part of the system.

Wired architectures are widespread, as they can implement a traditional clock-sharing strategy (e.g., [4]), allowing for the more straightforward design of a multi-node system with synchronized nodes. However, when considering a scenario with multiple sensing nodes placed in distant body areas along the entire patient body, the presence of wires would restrict the usability of the product in clinical contexts. Hence, there is a need to develop innovative platforms; however, a significant technical challenge lies in designing these systems to be completely wireless and wearable in order to simplify their integration into patients’ daily life activities. Indeed, the wireless feature is a core point for promoting the use of these platforms in daily life scenarios; at the same time, however, each innovative architecture needs to undergo specific data quality assessments in order to certify its comparable performance concerning signals synchronization with respect to traditional wired in-lab equipment in which synchronization is achieved by design using wires.

Authors have studied this research topic in previous works; for example, an innovative wireless acquisition platform was presented [5,6]. The present study is an extended version of the one described in [6]. The studied platform is designed to be exploited for continuous monitoring applications; its main feature is its be multi-purpose nature, meaning that users can attach any kind of analog transducer to the sensing nodes and that the system is able to synchronize the acquisitions and transfer data to an external data collector. Specifically, the platform is based on a custom architecture made up of two main software components: an embedded synchronization engine able to provide a common time base to the sensing nodes required by the application, and a data transmission engine that is able to flush data collected during a previous acquisition session towards an external gateway in order to free up memory for the following session. In order to implement the two communication engines, the proposed software architecture exploits the 2.4 GHz radio antenna present in the selected hardware (the nRF52840 SoC chip by Nordic Semiconductor [7]). Synchronization is addressed with a tailored low-level driver that directly drives the antenna hardware and generates a custom wireless network used to manage the packet exchange in order to share a common clock among the different sensing nodes. Data transmission is addressed by exploiting the SoftDevice provided by Nordic Semiconductor, a pre-compiled and linked binary software component that manages the implementation of different kind of standard wireless protocols, which is designed with the aim of offering an abstract yet adaptable interface suitable for the development of BLE applications [8].

This research work is carried on as an assessment and validation of the performance of the overall custom software architecture in terms of its synchronization precision with respect to the synchronization engine and the throughput/transmission rate with respect to the data transmission engine. The aim is to evaluate and improve the quality of the collected physiological data by selecting the best-performing network settings and to evaluate the correlation between the gateway positioning and the data flushing speed.

Based on a literature analysis, several different validation strategies concerning wireless synchronization engines emerge. Römer et al. have provided an interesting and useful overview of this research topic [9], describing in detail both the technical issues and the various solutions that might be implemented. The most traditional approach is direct measurement of the time synchronization between the two elements that need to be synchronized. In this case, a time-logger is required in order to collect time-shift data from the different network elements (e.g., [10]). This solution allows a continuous diagnostic evaluation to be implemented which may be run even in real-time during working operations, as one of the sensing nodes can play the role of the time-logger. A drawback is that this method requires a supplementary data processing task in order to analyze time-shift data in real-time, which must be run simultaneously with data acquisition. An alternative approach is the indirect measurement method, where the measurement of a variable not directly related to a time quantity is required. In this case, an external analyzer device is needed, with the aim of recording observable events from which it is possible to derive time-shift information. Examples include a pin toggling task or the evaluation of different power consumption profiles (e.g., [11]). This solution avoids increasing the computational load of the platform; however, a drawback is that it usually requires the exploitation of additional in-lab hardware components such as the specific analyzer, limiting the feasibility of the diagnostic evaluation to the factory production stage.

Similarly, several approaches have been proposed in the literature concerning the assessment of data transmission engines based on BLE in order to ensure reliability and efficiency across various applications. Numerous studies have investigated different aspects of BLE-based data transmission in order to understand its limitations and improve its capabilities [12]. Gomez et al. [13] focused on optimizing the piconet size, i.e., the maximum number of peripheral nodes that can be linked to a single central node. Because there is no predefined limit on the number of connections in the official BLE specifications, this parameter can be optimized by tuning the BLE software stack, such as its connection parameters, or specific hardware features such as the memory capacity of the BLE transceiver or the hardware antenna layout. Liu et al. [14] focused on optimizing the power consumption, correlating it with specific BLE advertising parameters (Adverting Interval, Scan Interval, Scan Window) and validating an analytical model of energy consumption in advertising mode that can be used to estimate battery life depending on the specific configuration. Buckley et al. [15] focused on optimizing the BLE connection’s maximum range and assessing the features that can influence it. According to their study, both hardware specifications (e.g., antenna layout and size) and environmental factors (e.g., the path loss, which is the energy waste experienced during communication and is strongly correlated with the distance between the two nodes) play a key role in the maximum range that can be achieved in a specific setup.

In this paper, considering the synchronization engine, we propose a direct method for implementing a diagnostic evaluation of the quality of data collected with the prototype platform described in [5,6]. The measurement setup involves the external gateway, which plays the role of the time-logger; two generic sensing nodes enrolled in the platform send timestamp data to the gateway with a fixed frequency, then the gateway computes the synchronization delay in real time. We repeated this evaluation while varying specific network parameters, specifically, the frequency of sending synchronization packets and the BLE Connection Interval, in order to study the possible correlation between the synchronization accuracy and the tuning of the network settings. Moreover, we computed two custom diagnostic metrics related to the synchronization packet loss issue due to the two simultaneous ongoing protocols, then compared them with the synchronization performance in order to study the possible correlation between packet loss and synchronization accuracy. Additionally, concerning the data transmission engine proposed in the prototype platform, we performed an empirical assessment to evaluate the throughput of the BLE stack provided by Nordic Semiconductor by simulating five different indoor home-monitoring scenarios. Because the BLE engine runs only between a single sensing node (which is in charge of sending the collected data from all the active nodes in the platform to the data collector) and the external gateway, we evaluated the data transmission rate when varying the distance between this node and the gateway, then assessed the effect of the presence of obstacles such as partition walls that can make the home environment a harsh one.

## 2. Materials and Methods

### 2.1. Hardware and Software Overview of the Platform

**Figure 1:**
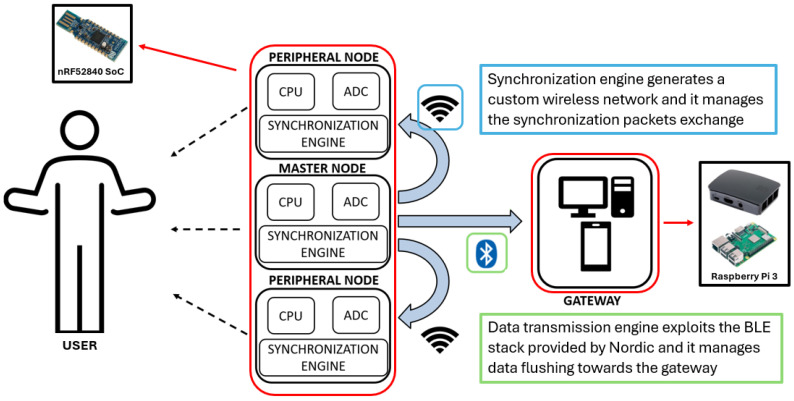
Overall platform overview. (1) Concerning the hardware components, each sensing node is based on the nrf52840 Dongle architecture provided by Nordic Semiconductor. The user can use the nodes to connect whatever analog transducer is useful for the specific application (multi-purpose feature). The external gateway acts as the data collector; in the proposed setup, we exploited a Raspberry Pi 3. (2) Concerning the software components, the platform relies on two engines: the synchronization engine, implemented with a custom wireless protocol and the data transmission engine, implemented using the standard BLE protocol.

The prototype wearable platform under analysis is a multi-node acquisition system based on a fully wireless software architecture (Figure 1), and is intended to ensure the required wearability in daily life scenarios [5,6].

**Figure 2:**
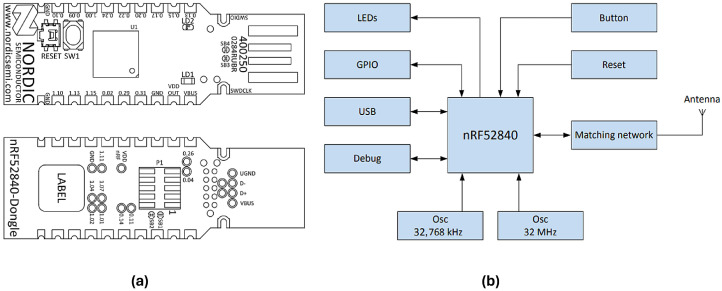
(**a**) Front and back views of the nrf52840 Dongle and (**b**) functional block diagram of the peripherals added to the original nrf52840 SoC chipset [16].

From a hardware point of view, we used the nrf52840 Dongle component by Nordic Semiconductors to design the sensing nodes of the platform (Figure 2). The nRF52840 Dongle is a compact and affordable USB breakout that offers support for ANT, 802.15.4, Zigbee, Thread, Bluetooth mesh, classic Bluetooth, BLE, and proprietary protocols operating with the supplied 2.4 GHz antenna (relying on the SoftDevice library software).

Together with the full availability of drivers and documentation provided by the manufacturer, its low cost makes this component very attractive in prototype applications. The breakout is based on the nrf52840 SoC chipset (built around a 32-bit ARM^®^ Cortex™-M4 CPU, Cambridge, UK), and the internal ADC can be paired with any kind of analog transducer. The number of sensing nodes depends on the specific application (based on the number of required bio-signals); the only theoretical limit is the piconet size of the BLE network, which is usually an order of magnitude larger than the requirements of most home monitoring applications. The large number of degrees of freedom available to the user enhance the multi-purpose features prioritised during technical development of the platform. We consider this to be the platform’s main strength, as the resulting flexibility allows for its exploitation in a wide range of different use cases and scenarios, both clinical and otherwise. For example, possible applications outside of clinical scenarios could include assessing the improvement of an athlete during physical training sessions or the real-time evaluation of athletes’ performance during long-term sporting competitions, as well as the development of innovative systems to monitor the stress level of employees in the working environment or to allow more complex human–computer interaction (HCI) applications.

Additionally, we introduced a data collection element into the hardware architecture in order to design a product able to perform continuous monitoring tasks. Indeed, without the possibility of periodically flushing their memory, nodes are forced to collect data only for a limited period of time (depending on the bio-signals sampling frequency required by the application) before having to overwrite the collected information. The Raspberry Pi 3 is an attractive device for developing gateway prototypes thanks to its versatile capabilities and affordable price. With its robust processing power, expandable memory options, and variety of connectivity interfaces, including Wi-Fi and BLE, it provides a solid starting point for rapidly prototyping projects in different fields ranging from IoT and robotics to home monitoring.

**Figure 3:**
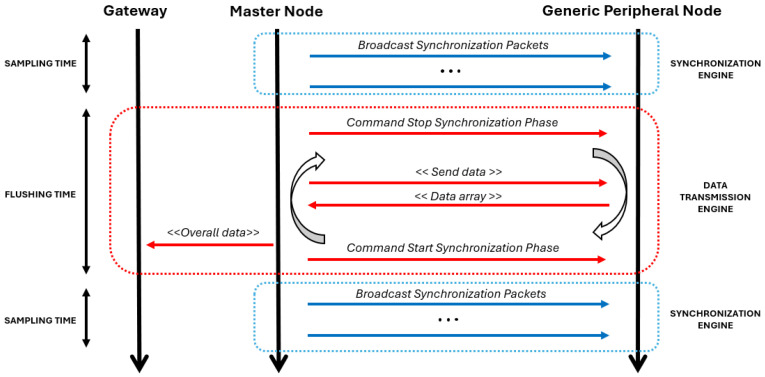
Dual-protocol approach during a normal acquisition cycle. The procedure starts with the synchronization engine, which sends synchronization packets for a specific time (the sampling time) by exploiting the low-level proprietary protocol. During this period, different nodes sample the required bio-signals synchronously. As soon as the sampling time expires, the synchronization engine and sampling phase are blocked and the data transmission engine starts its lifecycle. First, the datastream collected from all of the nodes is channelled into the MN, then the stream is flushed towards the external gateway by exploiting the standard BLE protocol. When the process is completed, the overall cycle starts again with a new sampling phase.

From a software architecture point of view, the main technical innovation is the dual protocol approach, which means that the platform establishes two concurrent wireless networks. Among all the sensing nodes enrolled in the network, one node is the most important, which is called the Master Node (MN), while all the others are generic Slave Nodes (SN). All the nodes are part of a BLE network, in which the MN is the BLE Central Node and the SNs are BLE Peripheral Nodes. This network is responsible for data transmission. Additionally, The MN is in charge of sharing its time base with all the others, broadcasting synchronization packets with a configurable frequency to create the synchronization network responsible for ensuring a synchronized sampling (Figure 3).

The first network is managed by the data transmission engine, and is based on BLE technology, a standard wireless protocol that allows for data transfer with an external gateway through extra-platform communication. The gateway is designed to be a generic commercial device owned by the platform end-user in a future engineering phase; thus, it should usually expose only standard APIs concerning wireless communication. Hence, there is need for a standard protocol to share the acquired data, despite heavy communication overhead that makes such communication slower than directly driving the hardware antenna as can be done in a proprietary wireless protocol.

The second network is managed by the synchronization engine, and is based on a custom wireless protocol that keeps the sensing nodes synchronized with each other during bio-signal acquisition. In this case, we are dealing with intra-platform communication, as we do not have to interface with external elements; thus, we are able to use a lighter proprietary protocol. This is the key point of the platform, as it becomes possible to share a common time base among all of the sensing nodes enrolled in the system in a faster and more precise way then using a standard protocol. Indeed, the aim of this network is to exchange synchronization packets while reducing communication overhead and latency as much as possible [5,6]. The quality of the physiological measurements obtained from the system relies directly on the precision of the synchronization engine. When nodes lack synchronization, the acquired signals are based on different time bases, resulting in significant time lag between acquisitions from different nodes and decreasing the overall data quality. In most biomedical scenarios, synchronization is typically considered to be achieved when physiological signals are time-shifted by less than 500 µs, assuming the sampling frequency of 1 kHz commonly used in biomedical applications. Therefore, in order to meet the strict data quality standards required by the healthcare context, the system must maintain synchronization delays that are as constant as possible, ideally within a few tens of microseconds. However, clock drift issues can cause these delays to exceed this threshold after few minutes of work, requiring a wireless synchronization engine able to provide consistently high performance over time.

### 2.2. Synchronization Engine Assessment

In a dual-protocol scenario, a significant challenge may arise in that the two protocols must operate concurrently while competing for the same shared radio hardware resources. In the proposed platform, although the engines running the two protocols work in separate time slots during normal operations (Figure 3), the risk of facing several packet loss occurrences is not negligible, as standard BLE requires periodic exchange of messages (0-byte data packets are exchanged in any case, even if the queue is empty) [17] and synchronization packets may be blocked due to the radio resources being already engaged by the BLE protocol. Consequently, this condition may downgrade both synchronization performance and overall data quality.

Typically, the level of interference between two protocols depends on how often and for how long each protocol monopolizes the antenna resources to complete its periodic message exchanges, preventing the other one from doing the same. In order to evaluate the impact of this issue, we carried out a quantitative assessment to study how synchronization performance varied under different network settings. We evaluated two different network variables affecting the radio occupancy time: the synchronization packet sending rate, concerning the occupancy time of the proprietary protocol, and the connection interval, concerning the occupancy time of BLE protocol (see Section 2.2.1).

Additionally, we designed two custom diagnostic metrics to quantify the packet loss resulting from the interference between the two protocols, then compared these metrics with the synchronization performance. An increase in packet loss is expected to correspond to poorer synchronization, and vice versa (see Section 2.2.2).

#### 2.2.1. Tuning of Network Parameters

In the analysis described in this section, we want to establish whether tuning the settings of the two networks (both the BLE and the custom one) affects the synchronization performance. The frequency of sending synchronization packets and the introduction of a dynamic adaptation in the BLE connection interval parameter are the two network variables that we tested. Both affect the overall amount of time for which the radio hardware resource is required by the two protocols.

Tuning different frequencies for sending synchronization packets impacts the overall time for which the synchronization protocol exploits the antenna; as the frequency rises, the resource utilization time increases as well. Thus, increasing the synchronization frequency could potentially result in a more significant interference grade and a higher probability of packet loss; on the other side, however, it can ensure more robust synchronization performance over time.

**Figure 4:**
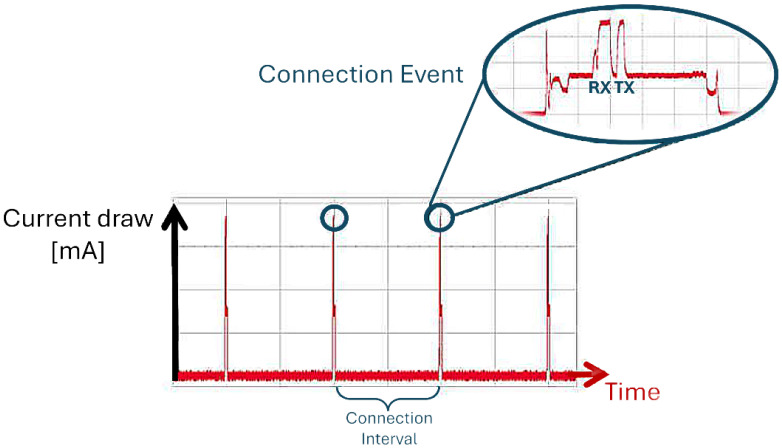
Two BLE devices exclusively communicate with each other over a designated channel at specific times. After a predefined timeout, the devices connect again on a new channel; channel switching is handled by the link layer of the BLE protocol stack. When data exchange occurs between the devices, this pairing is referred to as a connection event. In the absence of application data for transmission and reception, the two devices exchange standard link layer data (0-byte data) to maintain the connection. The time shift between two consecutive connection events, measured in units of 1.25 ms, is called the connection interval. This interval can vary from a minimum value of 6 (equivalent to 7.5 ms) to a maximum of 3200 (equivalent to 4.0 s) [18].

The connection interval parameter is the time interval between two consecutive connection events. A connection event is a periodic data transfer event between a BLE central node and a BLE peripheral node, and is performed with a specific frequency, which is the connection interval (Figure 4). According to the official BLE specifications, acceptable values can vary between 7.5 ms and 4 s, and the choice should be made by weighing the trade-off between data throughput and radio occupancy time (and consequent higher power consumption, another important issue, which is not addressed in this study). Selecting a higher connection interval value reduces the radio’s usage over time, lowering the probability of interference and packet loss, but also results in decreased data throughput, i.e., a reduced data transmission speed, with the reverse also being the case.

**Figure 5:**
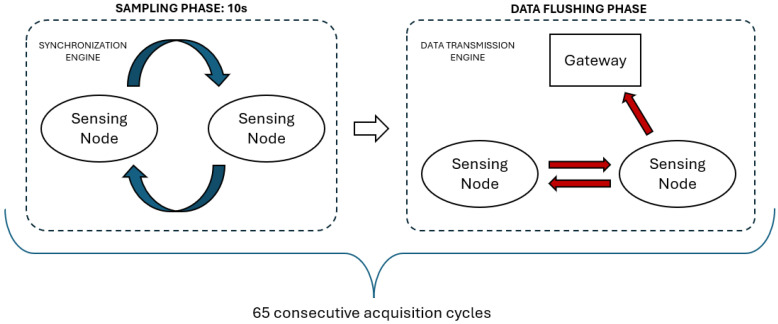
Overview of the experimental setup. We performed 65 acquisition cycles, first collecting data during a sampling phase of 10 s and then making the information available to an external gateway in the data flushing phase. This protocol was repeated for each of the seven tested synchronization frequencies as well as for the evaluation of the connection interval dynamic adaptation.

First, we performed quantitative measurements to establish the optimal synchronization frequency for the proprietary software architecture under analysis. We exploited two sensing nodes during the ordinary platform working scenario, as described in Figure 3. We tested seven different synchronization frequency values in a range from 10 Hz to 70 Hz (in steps of 10 Hz), and for each one we performed 65 acquisition cycles, first running the synchronization engine during the sampling phase with a fixed duration of 10 s and then the data transmission engine during the data flushing phase (Figure 5). The two nodes were placed 70 cm apart one from one another on a desk in order to simulate the average distance between two sensors on different body areas. In order to assess the synchronization performance, during the sampling phase we collected the timestamp at which the internal ADC samples the input for both nodes using a standard 1 kHz sampling frequency. The clock drift issue would lead these two timestamps to become further and further apart in time; however, the synchronization engine periodically realigns the two nodes, with the frequency depending on the synchronization frequency. During the data flushing phase, the collected timestamp data are then aggregated by the gateway, which is located 1 m from the master node, and an algorithm measures the real-time time shift between the two nodes. The optimal synchronization frequency is assessed as the one that provides the lower time shift considering both the average value and the variability of the measurements. Python language (version 3.7.0) was used to develop the elaboration algorithm.

Additionally, after assessing the optimal synchronization frequency for the architecture, we carried out a test to dynamically tune a parameter specific to the BLE protocol. We tested the tuning of the BLE connection interval value at run-time during platform operation, setting a higher value (180 ms) during the synchronization phase and a lower value (7.5 ms) during the flushing phase. The former is expected to result in a decrease in the interference probability, while the latter is expected to increase the data transmission speed towards the external gateway. We fixed the synchronization frequency to the optimal value according to the outcomes of the previous analysis, then performed 65 acquisition cycles with a sampling phase of 10 s both with and without the adaptive strategy (Figure 5). Also in this case, we collected timestamp values during the synchronization phase and measured the real-time time shift between the two nodes using an algorithm running on the gateway. The position of the two nodes on the desk remained the same at 70 cm apart, and the gateway was again located 1 m from the master node.

The objective of this analysis was to achieve a balance between low interference and reliable synchronization. The expected result was the identification of the best-performing network configuration that minimizes the average synchronization delay among sensing nodes while enhancing synchronization performance, consequently improving the overall data quality provided by the proposed wearable system.

##### Statistical Validation

We conducted a statistical analysis in order to determine whether there was a significant difference in terms of the delays between the explored range of synchronization frequencies and the two tested BLE configurations. We evaluated two distributions concerning the BLE configuration analysis and seven distributions concerning the frequency range analysis.

- Concerning the BLE configurations study, we first subjected the two distributions under analysis to the Kolmogorov–Smirnov test, a nonparametric test that can be used to test whether a sample comes from a given reference probability distribution. Our aim was to understand whether the two distributions were normally distributed. After that, we subjected the same two distributions to the Mann–Whitney U test, used to assess whether two groups come from the same population. This test does not assume any specific distribution, such as a normal distribution, for calculating test statistics and p values. Our aim was to understand whether there was a significant difference in terms of delay between the two tested BLE configurations.
- Concerning the frequency range study, we first subjected the seven distributions under analysis to the Kolmogorov–Smirnov test, with the aim of understanding whether the seven distributions were normally distributed. After that, we subjected the same seven distributions to the Kruskal–Wallis test, which compares three or more groups in terms of a quantitative variable, and can be seen as an extension of the Mann–Whitney U test. Additionally, we performed a post hoc analysis with Dunn’s test, usually used to uncover specific differences between three or more group means when an analysis of variance (in this case, the Kruskal–Wallis test) leads to a significant result. Our aim was to understand whether there were significant differences in terms of delay between the seven tested synchronization frequencies.

##### Experimental Design

- Frequency-Range Protocol
  We exploited two sensing nodes and a gateway located 1 m away from the MN. The two nodes were placed on a desk 70 cm apart one from the other in order to simulate the average distance between two sensors placed in different body areas. We tested seven different synchronization frequency values in a range from 10 Hz to 70 Hz, in steps of 10 Hz. For each frequency, we performed 65 acquisition cycles with a fixed duration of 10 s in order to compare the distributions of the synchronization delay (computed processing timestamp data).
- Connection Interval Protocol
  We exploited two sensing nodes and a gateway located 1 m away from the MN. The two nodes were placed on a desk 70 cm apart one from the other in order to simulate the average distance between two sensors placed in different body areas. We fixed the synchronization frequency to the optimal value according to the outcomes of the previous analysis. We tested two different BLE configurations, one with an adaptive connection interval value and the other with a fixed value. For each configuration, we performed 65 acquisition cycles with a fixed duration of 10 s in order to compare the distributions of the synchronization delay (computed processing timestamp data).

#### 2.2.2. Diagnostic Metrics for Packet Loss

In the analysis described in this section, we compare the differences in synchronization performance resulting from the tuning described in the previous section with two custom diagnostic metrics related to packet loss. The aim is to confirm that there is a correlation between the synchronization delay among two nodes and the interference due to the radio hardware occupancy.

Both the synchronization engine and the data transmission engine involve all of the node in the platform; indeed, both the MN and all the SNs concurrently implement the synchronization and the BLE protocols. While the synchronization protocol only requires that the MN send data and the SNs elaborate the received synchronization packets, the BLE protocol requires that the Central Node and the Peripheral Nodes both send and receive data during each connection event. Therefore, protocol interference may occur during synchronization packet transmission (on the MN side) as well as during synchronization packet reception (on the SN side). Hence, we elaborated two specific diagnostic metrics to quantify the packet loss issue, one referring to the packet sender (the MN) and the other one to a generic packet receiver (each SN).

During a normal acquisition cycle, considering a platform with just two nodes for the sake of simplicity, when the MN sends a synchronization packet to the SN it also updates an incremental packet counter and shares this counter with the SN in the packet payload (together with its own time base, which is required to allow the other nodes to synchronize with it). When the SN elaborates the received packet, it updates its own time base to align with the MN and saves the packet counter it has just received. If there is no packet loss issue, both the MN and SN are expected to update their packet counters concurrently.

**Figure 6:**
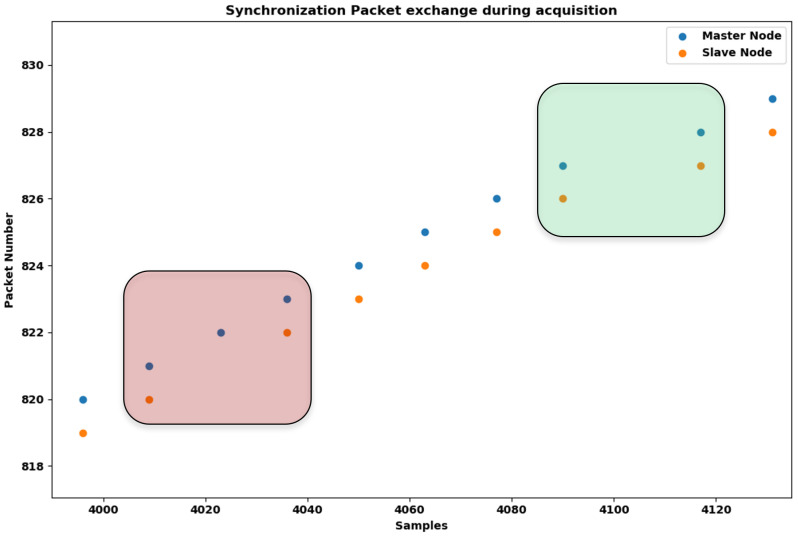
Examples of the packet loss issue from both the MN and SN sides (the green and red boxes, respectively). The proposed diagnostic metrics, MRF and SPL, aim to quantify this phenomenon [6].

Figure 6 shows a graphical explanation of the packet loss issue from both the MN and SN sides. The green and red boxes respectively identify the two critical situations we want to quantify with the two metrics. Master Radio Failure (MRF) is the first metric; it refers to the situation in which the MN fails to send the planned synchronization packet due to the ongoing BLE protocol. In this case the MN is forced to skip the sending process, and neither the MN nor SN updates the packet counter at the expected time (the green box). The second metric, the Slave Packet Loss (SPL), refers to the situation in which the MN sends the planned synchronization packet as expected, but the SN fails to receive it. In this case, the MN updates the packet number count while the SN does not (the red box). In both cases, a packet loss issue leads to overall worsening of the synchronization of the bio-signal acquisitions.

We computed these metrics to express the packet loss percentage for each of the seven tested synchronization frequency values and compared the outcomes with the synchronization performance resulting from the investigated frequency ranges. Along with timestamp data, we collected packet counter data from the MN and SN during the same acquisitions described above (Figure 5), with 65 acquisition cycles for each evaluated synchronization frequency and the two nodes placed 70 cm apart on a desk. As both metrics represent a percentage of the packet loss, we calculated them for all of the investigated frequency ranges and for all acquisition cycles by comparing the actual number of packets sent (MRF parameter) or received (SPL parameter) obtained from the nodes to the theoretical amount we expected to receive considering the synchronization frequency and the fixed sampling time (10 s). The aim of the subsequent analysis was to confirm a possible correlation between the variation in synchronization performance and the packet loss phenomenon.

Additionally, we performed experimental data collection on a population of fifteen healthy subjects in order to validate the proposed prototype platform for a clinical use case. The population age ranged from 23 to 57, with an average value of 32.3 years and a standard deviation of 9.7 years. The Politecnico di Milano Ethics Committee approved the study (opinion n. 44; 13 December 2023), and the volunteers were asked to provide written informed consent. We used the platform with the basic configuration consisting of two nodes, placed on the subject’s chest to collect ECG data and on the wrist to collect PPG data. During these acquisitions, we collected packet counter data from both the MN and the SN, based on which we computed the proposed diagnostic metrics. During this experimental data collection process, we set the optimal synchronization frequency resulting based on the previous tuning and performed ten acquisition cycles for each subject. The subjects were then required to perform a moderate physical activity task on a treadmill for 3 min, after which we performed other ten acquisition cycles. The two nodes were attached to the body at a distance of approximately 70 cm from each other. For each subject, we computed the diagnostic metrics for all the acquisition cycles (20 in total). The comparison among the values of the two diagnostic parameters collected during an on-field validation and during the previous acquisitions on the laboratory desk is interesting, as it can highlight possible differences between the ideal setup and a real-world scenario.

##### Experimental Design

- In-Lab Protocol
  We exploited two sensing nodes and a gateway located 1 m away from the MN. The two nodes were placed on a desk 70 cm apart one from the other in order to simulate the average distance between two sensors placed in different body areas. We tested seven different synchronization frequency values in a range from 10 Hz to 70 Hz, in steps of 10 Hz; for each frequency, we performed 65 acquisition cycles with a fixed duration of 10 s in order to compare the distributions of the diagnostic metrics values (computed processing packet loss data).
- On-Field Protocol
  We exploited two sensing nodes and a gateway located 1 m away from the MN. The two nodes were attached to the body of a patient, either on the subject’s chest to collect ECG data or on the wrist to collect PPG data, 70 cm apart one from the other. We fixed the synchronization frequency to the optimal value and implemented the connection interval run-time adaptation according to the outcome of the previous analysis. For each subject, we performed 20 acquisition cycles with a fixed duration of 10 s in order to compare the distribution of the diagnostic metric values (computed processing packet loss data).

### 2.3. Data Transmission Engine Assessment

In this section, we provide a detailed analysis of the data transmission engine. This software module relies on the BLE network established among all the nodes in the platform and the external gateway. The network is built by exploiting the BLE stack provided by Nordic Semiconductors. Specifically, we implemented the BLE GATT Nordic UART Service (NUS) in both the nodes and in the gateway. This service, which is a proprietary BLE service used to send and receive data over BLE, acts as a bridge to the UART interface, simulating a serial communication over BLE technology. This is very suitable in our scenario, as the aim of the network is to transfer a stream of data.

As previously described, BLE specifications require periodic message exchange even if there are no data to be flushed; therefore, the connections between the BLE Central Node (the MN) and all the other BLE Peripheral Nodes (the SNs and the gateway) are always running. These are managed by the Nordic SoftDevice. When the sampling time expires, the data transmission engine starts working and takes control of the BLE network. At this point, the data transmission engine handles two different tasks:

1. Data Aggregation: The MN stops sending synchronization packets over the custom wireless network and starts scanning the platform via BLE, asking each SN in turn for the data collected during the sampling phase that has just ended. All of the SNs send the signal track they acquired to the MN, and the MN stores all the information in its internal memory.
2. Data Flushing: The MN flushes the entire dataset towards the external gateway. It sends its own acquisition first, then sends the data streams collected from all the other SNs active on the platform in turn. As soon as the last packet is sent, the data transmission engine is stopped and the synchronization engine starts again, with the MN ready to perform a new sampling phase and kick off a new acquisition cycle broadcasting synchronization packets.

In the scenario we are considering in this study, the most important feature that the data transmission engine must provide is the speed of data transfer. The connection’s reliability is ensured by the use of the BLE protocol, an established and now widely validated technology. Because the proposed platform works with a time-slot architecture (Figure 3), faster data transfer means that a new acquisition can be started sooner. This is a key point, as a possible metric for assessing the platform’s performance from a functional point of view might be the ratio between the sampling time and the total acquisition time (sampling plus data transfer, which for all intents and purposes can be considered as a “blind period”).

**Figure 7:**
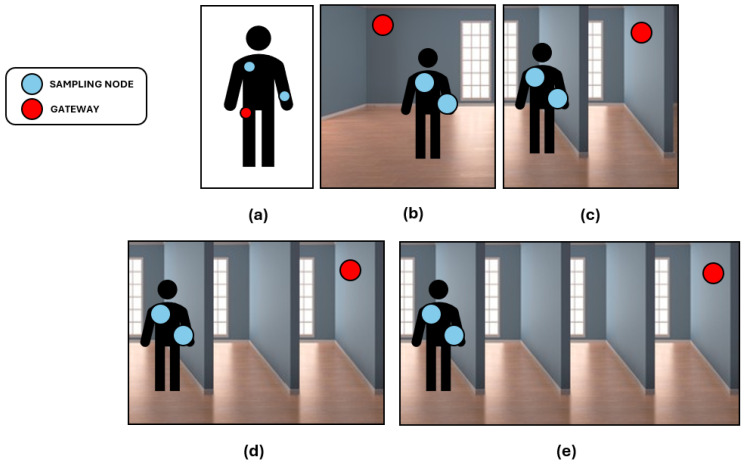
Exemplification of the real-world scenarios simulated by changing the distance between the gateway and the sensing nodes. During data collection, the sensing nodes were always 70 cm apart one from the other, placed on a desk and without any physiological input signal, while the gateway was placed at increasing distances to simulate several scenarios. (**a**) Here, the gateway is a custom wearable device or a commercial device such as a smartphone, and is placed 70 cm apart from the nodes. (**b**) The gateway is an environmental device placed 4 m from the nodes in the same room, ensuring line-of-sight. (**c**) The gateway is an environmental device placed one room away from the sensing nodes, leading to a non-line-of-sight scenario due to a partition wall. The gateway is placed 6 m from the nodes, with the wall as an obstacle in the middle. (**d**) The gateway is an environmental device placed two rooms away from the sensing nodes, leading to a non-line-of-sight scenario due to multiple partition walls. The gateway is placed 8 m from the nodes, with multiple obstacles in the middle. (**e**) The gateway is an environmental device placed three rooms apart from the sensing nodes, experiencing an even more extreme non-line-of-sight scenario due to multiple partition walls. The gateway is placed 10 m from the nodes, with multiple obstacles in the middle.

In order to quantify the throughput achieved with the proposed data transmission engine, we designed an experimental setup with two sampling nodes and the gateway. Although many variables may be considered in the optimization of the throughput of a BLE network, we decided to focus on the distance between the BLE nodes. It is well known from the literature that BLE throughput is inversely related to the distance; however, we wanted to experimentally quantify the performance of the specific BLE stack provided by Nordic Semiconductor in interfacing with the Linux BLE stack implemented in the gateway. We tested the architecture with a simulation of five different real-world scenarios involving a platform composed of two nodes, one on the chest and one on the wrist, approximately 70 cm apart one from the other, and a gateway that could be placed at different distances. These scenarios are summarized in Figure 7.

We maintained the sampling nodes in the same position as the other conducted tests, on a desk 70 cm apart one from each other without any physiological input signal, but changed the gateway’s position relative to the MN, which is the only node in the network able to communicate with the external data collector via BLE. The first scenario, with the gateway 70 cm from the MN, exemplifies a situation in which the gateway is a custom wearable device or commercial device in proximity to the subject, such as a smartphone inside its pocket. The second scenario, with the gateway 4 m from the MN, exemplifies a situation in which the gateway is an environmental device such as an anchor placed in a room, while the subject is roaming inside. The third scenario, with the gateway 6 m from the MN with a partition wall between the two, exemplifies a situation in which the gateway is an environmental device and the specific home environment is particularly harsh due to limited presence of open spaces. The fourth and fifth scenarios, with the gateway 8 m and 10 m from the MN, respectively, with multiple partition walls between the two, exemplify situations in which the gateway is an environmental device placed in a room even further away from the one in which the sensing nodes are performing acquisition.

For each scenario, we performed 100 acquisition cycles with a sampling phase of 10 s, first running the synchronization engine and then the data transmission engine. In order to assess the throughput performance, during the data transmission phase, the MN stores the time spent in each step (three in total) of the transfer phase:

- SN flushes data to MN
- MN flushes its data to GATEWAY
- MN flushes data from SN to GATEWAY

We collected these timings through a serial debug connection with the MN for each performed acquisition cycle, then compared the outcomes across the different scenarios. The aim of this analysis was twofold: to evaluate the impact of selecting one type of gateway over another (wearable or environmental) in terms of data throughput performance, and to determine which is currently the best performance the proposed platform can assure in terms of the ratio between the active (sampling) and inactive (data transmission) operation times.

#### Experimental Design

We exploited two sensing nodes and a gateway located at a variable distance from the MN. The two nodes were placed on a desk 70 cm apart one from the other in order to simulate the average distance between two sensors placed in different body areas. We tested five different distances between the gateway and the MN (70 cm, 4 m, 6 m, 8 m, 10 m); for each scenario, we performed 100 acquisition cycles with a fixed duration of 10 s in order to compare the distributions of the time required by the three steps of the data transmission phase (computed by measuring the time needed to complete each BLE transmission task).

## 3. Results

We conducted a series of experimental measurements aimed at evaluating how adjustments in specific network settings could impact synchronization performance. Our analysis focused on two distinct network variables: the frequency of synchronization packet transmission from the MN to the SN, varying from 10 Hz to 70 Hz in steps of 10 Hz, and the implementation of a dynamic adaptation in the connection interval value. For the latter, we tested a lower value than the standard one in order to minimize protocol interference during the sampling phase. Subsequently, we analyzed the correlation between our custom diagnostic metrics quantifying packet loss between the two test nodes and synchronization performance. To further validate our findings, we repeated this assessment in a real-world biomedical scenario, then compared the outcomes with the ones obtained from our in-lab measurements. Additionally, we evaluated data transmission performance by simulating five different real-world scenarios and measuring the total time taken by the MN to flush the entire platform data stream to the external gateway via BLE and start the next sampling phase.

### 3.1. Tuning of Network Parameters

Concerning optimization of the custom protocol settings in order to reduce the synchronization delay, we tested seven different synchronization frequency values; for each one, we performed 65 acquisition cycles with a sampling phase of 10 s. In each sampling phase, we collected timestamps data from the two nodes with a frequency of 1 kHz, while the gateway computed the synchronization delay distribution time-by-time. The synchronization delay was computed as the difference between two corresponding timestamps.

**Figure 8:**
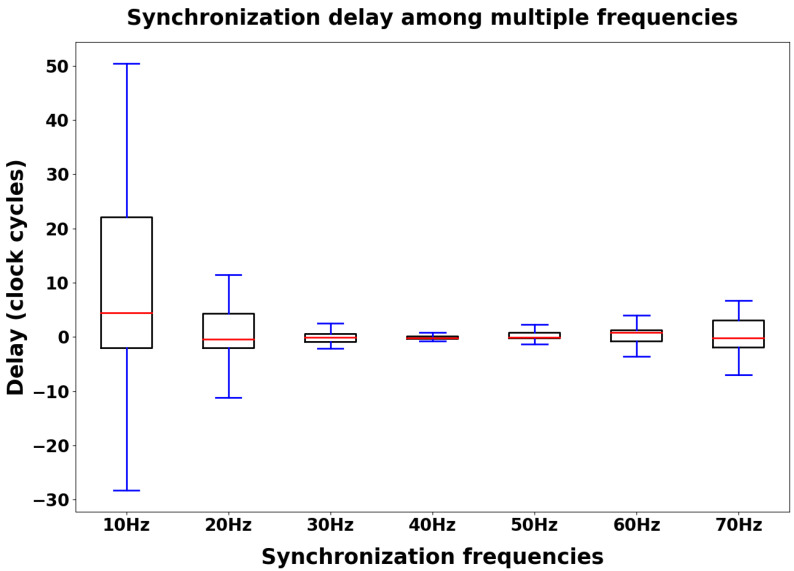
Distribution of synchronization delay among multiple synchronization frequencies. We collected timestamp data from the two sampling nodes for each value of the investigated frequency range, then computed the delay as the difference between two corresponding timestamps [6].

**Figure 9:**
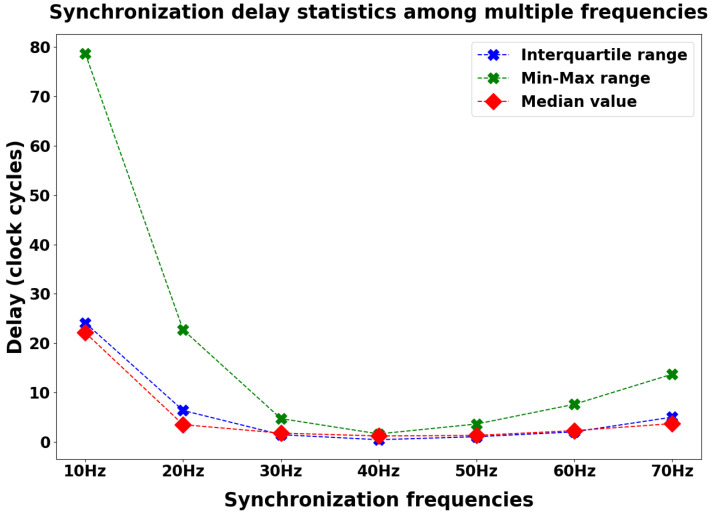
Statistical features (median value, min–max range, and interquartile range) concerning the synchronization delay distributions among multiple synchronization frequencies.

Figure 8 shows the box plots related to the synchronization delay distributions collected during the measurement sessions. For each synchronization frequency, Figure 9 shows the behavior of three statistical features (the median value, the min–max range, and the interquartile range) computed on the synchronization delay distributions (considering the absolute value). The synchronization frequency of 40 Hz has the best performance in terms of lower synchronization delay between the two nodes (1.18 ticks median value, 1.60 ticks min–max range, and 0.42 ticks interquartile range). Because the architecture is based on a 16 MHz clock cycle, considering the International System of Units (SI), each tick is equivalent to 62.5 ns.

Concerning the optimization of the BLE protocol settings to reduce the synchronization delay, we evaluated the introduction of a dynamic adaptation in the connection interval value. A larger value means that the BLE protocol exchanges packets with a lower frequency, keeping the radio busy for a shorter total time while slowing down BLE communication (and vice versa). The idea is to set a higher value (180 ms) during the sampling phase in order to affect the amount of radio resource needed by the custom protocol as little as possible, then a lower value (7.5 ms) during the data transmission phase to speed up the process of data flushing towards the gateway.

**Figure 10:**
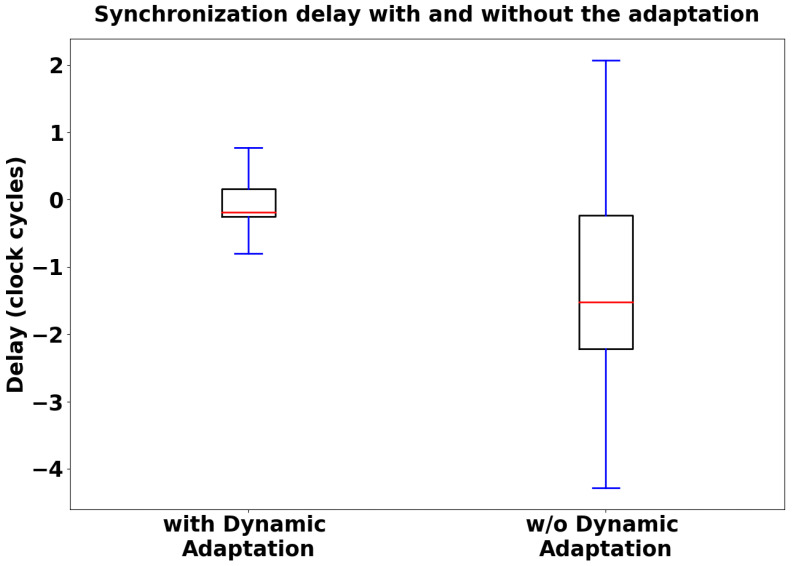
Distributions of synchronization delay with and without dynamic adaptation of the connection interval parameter; 40 Hz was used as the synchronization frequency [6].

We set the synchronization frequency at 40 Hz according to the outcomes of the previous analysis (Figure 8 and Figure 9) and collected timestamp data from the two nodes during 65 acquisition cycles with a sampling phase of 10 s. We performed two different measurement sessions, one with the adaptive strategy running and the other without (i.e., keeping the value fixed at the lower value for the full time). Computation of the synchronization delay distributions followed the same rules described in the previous analysis. As expected, as shown in Figure 10, the synchronization error during the sampling phase was minimized with the dynamic adaptation (1.18 ticks vs. 3.05 synchronization delay median value, considering the absolute value of the distributions).

#### Statistical Validation

We conducted a statistical analysis in order to determine whether there were significant differences in terms of delay between the explored synchronization frequency range and the two tested BLE configurations (Figure 8 and Figure 10). We evaluated two distributions for the BLE configuration analysis and seven distributions for the frequency range analysis.

Concerning the BLE configuration study, we performed the Kolmogorov–Smirnov test on the two distributions in order to assess whether they were normally distributed. The results in terms of the statistics and *p*-value are reported in Table 1.

The two distributions are not normally distributed, as the *p*-value is below the fixed significance level (0.05). Thus, in order to assess the statistical difference, we performed a non-parametric test, specifically, the Mann–Whitney U test, achieving a *p*-value of 0.00076. As the *p*-value obtained from the test was significant (<0.05), the two distributions differ significantly from each other.

Concerning the frequency range study, we performed the Kolmogorov–Smirnov test on the seven distributions in order to assess whether they were normally distributed. The results in terms of the statistics and *p*-values are reported in Table 2.

Among the seven distributions, only one (30 Hz) is normally distributed. Thus, in order to assess the statistical difference, we performed a non-parametric test, specifically, the Kruskal–Wallis test, achieving a *p*-value of 0.008. As the *p*-value obtained from the test was significant (<0.05), we can assess that at least one frequency is different from the others in terms of the synchronization delay distribution. Subsequently, we performed Dunn’s test, which is used to identify pairwise differences among different distributions when statistically significant differences are detected between distributions in the omnibus test. The results in terms of *p*-values are reported in Table 3.

### 3.2. Diagnostic Metrics for Packet Loss

**Figure 11:**
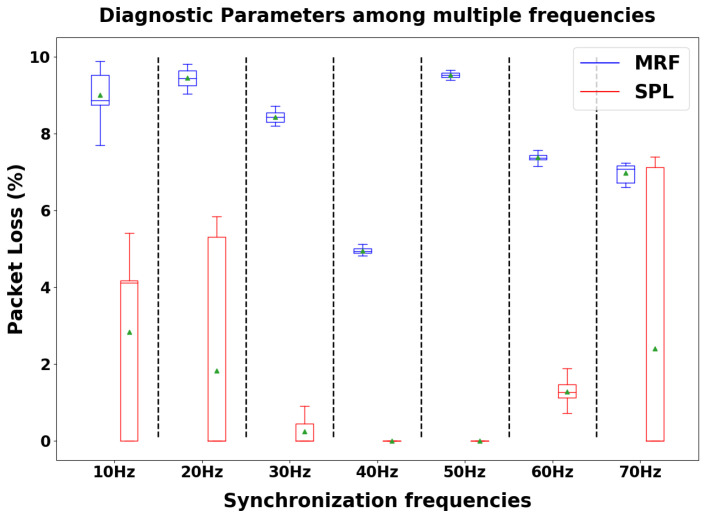
Distributions of network diagnostic metrics (in terms of loss percentage) over the investigated frequency range. A frequency of 40 Hz leads to the minimization of packet loss [6].

In this further analysis, we evaluated our custom diagnostic metrics for quantifying the packet loss issue. The aim of these metrics is to confirm a correlation between the variation in synchronization performance and the variation in radio hardware occupancy time. We collected packet loss data for each synchronization frequency during the same measurement sessions described above with regard to the timestamp data, then computed the percentage distributions of the packet loss by comparing the number of packets that were actually sent (the MRF parameter) or received (the SPL parameter) with the theoretical expected amount. Figure 11 shows the results collected by computing the diagnostic metrics in the investigated frequency range.

Consistent with the previous results, we achieved minimization of the metrics quantifying packet loss when setting a synchronization frequency of 40 Hz (4.95% loss percentage as the median value for the MRF parameter, 0% loss percentage for the SPL parameter). In support of the hypothesis that the radio occupancy time affects performance, 40 Hz is precisely the synchronization frequency that also minimizes the synchronization delay according to the analysis shown in Figure 9.

Additionally, we performed experimental data collection on a population of fifteen subjects in order to validate the proposed prototype platform in a clinical use case. On this occasion, we had the opportunity to collect data on packet loss during a field application. For each subject, we performed a total of 20 acquisition cycles while the subject was sitting on a chair, 10 before and 10 after a 3-min pause when the subject was required to perform moderate physical activity on a treadmill. We aggregated the two sessions and evaluated a single dataset of 20 acquisition cycles per subject, then computed the distributions of the metrics in terms of the packet loss percentage as described above.

**Figure 12:**
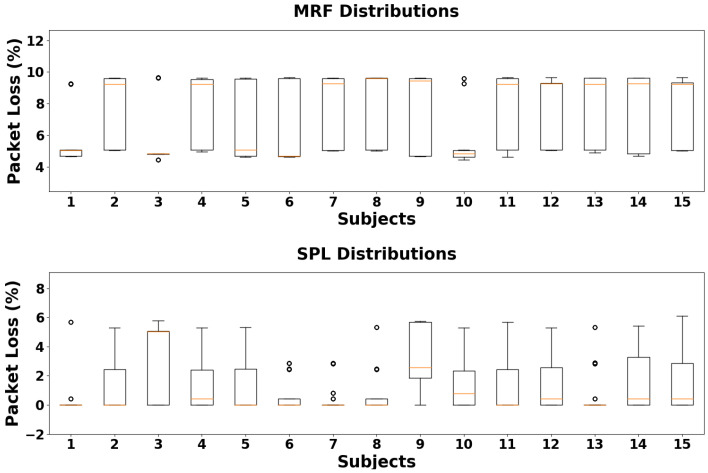
Distributions of network diagnostic metrics (in terms of loss percentage) for each subject in the study population (20 acquisition cycles each). The MRF parameter shows stability over the time in a specific range of values, while SPL shows a greater intra-population variability.

Figure 12 shows the results collected by computing the diagnostic metrics for each subject. Concerning the MRF parameter, the performance over time is stable regardless of the specific subject, with the packet loss falling between 4.43% and 9.67%. Concerning the SPL parameter, the data show more significant variability from subject to subject, with the loss falling between 0% and 6.09%.

### 3.3. Data Transmission Engine Assessment

As a final stage, we conducted a series of experimental measurements aimed at quantifying the throughput achieved with the proposed data transmission engine in terms of seconds needed to deliver the complete data flushing phase. We used the platform with the basic configuration of two sampling nodes plus the gateway, with the sampling nodes in a fixed position 70 cm apart one from the other and the gateway gradually moved farther from the MN. We tested five different scenarios with increasing distance; for each scenario, we performed 100 acquisition cycles with a sampling phase of 10 s while computing the distributions of the time required by the MN to deliver each step of the transfer (three steps in total).

**Figure 13:**
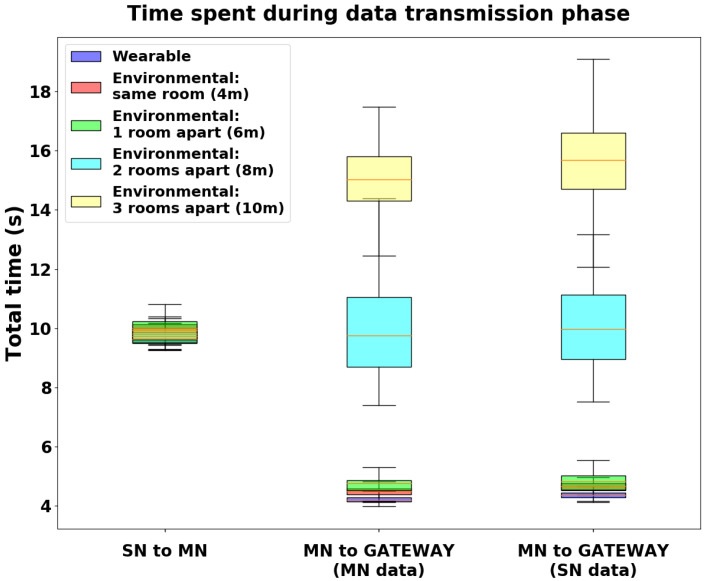
Distributions of the time required to carry out the three transmission steps for each tested scenario. The data flushing time increases progressively in the harsher home environments.

Figure 13 shows the aggregated results, with the distributions of the time required by the three steps for each tested scenario. The SN-to-MN communication maintains stable performance across the different scenarios (9.64 s, 9.94 s, 10 s, 9.73 s, and 9.82 s, respectively, concerning the median values). The MN-to-GATEWAY communication (split into two steps, MN data flushing and SN data flushing) grows progressively worse in the harsher home environments (4.2 s, 4.49 s, 4.77 s, 9.75 s, and 15.02 s, respectively, for the first step and 4.36 s, 4.66 s, 4.82 s, 9.98 s, and 15.68 s, respectively, for the second step, both concerning the median values).

As a further outcome, we computed a custom parameter, the Efficiency Index, to quantify the ratio between the sampling time (which was always kept constant at 10 s during all the measurements) and the the total acquisition time (the sampling time plus the data flushing time). This parameter can be considered a measurement of the platform’s efficiency, as higher values indicate a shorter blind period. Table 4 shows the value of this metric for each of the tested scenario, along with the mean and standard deviation values of the distributions. The parameter’s value is calculated by dividing the sampling time (10 s) by the mean value of the total acquisition time for each scenario.

## 4. Discussion

In recent years, extensive research has focused on evaluating the quality of data from wearable platforms. Multi-node wireless systems require highly accurate wireless synchronization engines to ensure that several sensing nodes stay synchronized during acquisition without the need for physical clock sharing, leading in turn to the need for quantitative performance assessments.

In this study, we propose a direct method to evaluate the synchronization performance provided by the innovative wireless monitoring platform presented in [5,6]. We computed the synchronization delay during the tuning of specific network parameters, namely, the sending frequency of synchronization packets and the BLE connection interval. In analyzing the frequency range under investigation, we found that exploiting the synchronization protocol with a frequency of 40 Hz led to optimal performance (Figure 8 and Figure 9). This frequency resulted in the lowest synchronization delay across the explored frequency range, striking a balance between synchronization consistency (achieved with higher frequencies) and minimal protocol interference (achieved with lower frequencies). Furthermore, dynamically adapting the connection interval value proved to be effective in further reducing the synchronization delay (Figure 10). These results support the hypothesis that slowing down BLE message exchange during the platform sampling phase can enhance synchronization performance. Considering the optimal performance achieved with a 40 Hz synchronization frequency and dynamic connection interval adaptation, this assessment suggests that the proposed platform may be well-suited for remote monitoring applications, as a median synchronization delay of 100 ns (converting collected clock ticks into ns) between two acquired bio-signals is acceptable in the healthcare monitoring context.

Furthermore, we conducted a statistical analysis in order to determine whether there was a significant difference in terms of synchronization delay between the explored synchronization frequency range as well as between the two tested BLE configurations. For both analyses, Kolmogorov–Smirnov tests (see Table 1 and Table 2) showed that the involved distributions were not normally distributed; therefore, we could perform only non-parametric tests. Concerning the two distributions evaluated in the BLE configuration analysis, we performed the Mann–Whitney U test, achieving a *p*-value of 0.00076. As the *p*-value obtained from the test was significant (<0.05), the two distributions significantly differed from each other in terms of median value, confirming the graphical results presented in the box plots in Figure 10. Concerning the seven distributions evaluated in the frequency range analysis, we performed the Kruskal–Wallis test, which allows for comparison of three or more distributions, achieving a *p*-value of 0.008. As the *p*-value obtained from the test was significant (<0.05), at least one distribution is different from the others in terms of synchronization delay. However, the Kruskal–Wallis test has a limitation in that it does not indicate which distribution(s) is (are) different from the others. Therefore, in order to deepen our analysis, after obtaining statistically significant Kruskal–Wallis results we selected a post hoc test, specifically Dunn’s test, to identify which specific pairs of distributions differed significantly from each other. The results of Dunn’s test (Table 3) showed that many comparisons led to distributions that were not significantly different from the others in terms of median values (*p*-value> 0.05). These results are consistent with Figure 8, as the delay distributions across the explored frequency range are similar with regard to the median value. Indeed, the main feature that makes the selected 40 Hz frequency the preferred one is the low variability in the collected measurements with respect to other frequencies, as shown by the interquartile range value and min-max value in Figure 9.

Additionally, regarding the custom diagnostic metrics that we developed (MRF and SPL), they showed a decreased packet loss percentage for both the MN and SN in our in-lab measurement sessions when employing a 40 Hz synchronization frequency (Figure 11). Occurrences of packet loss appear to be more frequent at higher frequencies, as expected, but also at lower frequencies, possibly due to synchronization packet timing being somewhat aligned with BLE message exchange. We performed a further data collection session concerning the custom diagnostic metrics directly in the field during the functional validation of the platform with a population of fifteen volunteers (Figure 12). The collected results show that the MRF parameter appears to be stable over the time within a specific range of values, although the performance is worse than the laboratory measurements. The field validation indicates that the sending frequency of synchronization packets and the BLE connection interval are insufficient to fully describe the packet loss phenomenon, as in the real-world scenario the MN experienced a larger packet loss percentage than in the laboratory setup, even though the network configurations were the same. Similarly, the SPL parameter also showed worse performance than in the ideal setup, though in this case weassessed a larger intra-population variability. This outcome may be explained considering that, by design, the SPL parameter includes not only the packets lost due to protocol interference but also those lost for other reasons, such as packets lost during travel between the two nodes. This phenomenon may be more common in real-world scenarios than in the lab environment, as the sensing nodes are attached to human bodies and excessive proximity to obstacles may prevent many synchronization packets from correctly reaching the SN.

As a last result, we proposed a quantitative assessment to evaluate the data transmission throughput ensured by the platform. We performed several measurement sessions to evaluate the impact of the distance between the external gateway and the MN during the data flushing phase via BLE (Figure 13). As expected, the collected outcomes show that the configuration with a wearable gateway ensures the best performance in terms of data transfer speed, while the configuration with an environmental gateway placed in the same room (or at most in a room adjacent to the sensing nodes) leads to similar outcomes. Performance exponentially degrades with the distance of the gateway from the node, leading to unacceptable results if the home environment is particularly harsh (e.g., the gateway is located two or three rooms away from the sensing nodes). These findings suggest that the environmental gateway should be preferred if possible, i.e., in a home environment that is compact and has many open spaces, as avoiding the need to add components to the wearable platform makes it less intrusive during the user’s daily activities. Furthermore, the custom Efficiency Index shown in Table 4 intuitively and quickly characterizes the scenario that the platform is working in, as it provides a value between 0 and 1, with 0 indicating an infinite data flushing time and 1 indicating instant data transfer. This index may be a valid choice for quickly assessing the characteristics of the home environment during installation of the monitoring system.

We have already identified a number of potential limitations of the proposed solution. One significant drawback could be the delay in detecting nodes disconnecting from the BLE network during the sampling phase, as decreasing the connection interval value slows down BLE message exchange, including keep-alive checks. However, it is worth noting that in a remote monitoring scenario, a delay of 180 ms to identify a disconnected node (the worst-case scenario with the proposed setup) is generally acceptable. Another constraint we observed is that if a 40 Hz synchronization frequency cannot be chosen, experimental results suggest opting for higher synchronization frequencies, despite this choice potentially leading to concerns around increased power consumption and reduced battery life of individual sensing nodes. Moreover, the proposed optimization analysis was tailored to the selected hardware (the nRF52840 SoC chip by Nordic Semiconductor, supporting the full Bluetooth 5.4 stack) and the custom implementation of the dual-protocol scenario (based on the nRF5 SDK version 16.0.0). The reproduction of the system might lead to different results if conducted with different hardware and software components. Concerning the data flushing stage, another drawback may be that the collected outcomes might force the use of multiple gateways if the home environment is particularly spacious or harsh, contributing to increased total cost of the system.

Future research directions to further improve the proposed platform’s performance are varied. Planned studies include exploring a broader range of synchronization frequencies and conducting a detailed investigation into power consumption across the frequency range under examination. Additionally, further studies will focus on evaluating the long-term stability of the proposed wireless architecture with an extensive field-testing period to evaluate the system’s reliability under typical working conditions. Moreover, to further improve the data transmission performance, one possible choice is to introduce a new wireless protocol for data transfer (such as Wi-Fi), which would allow a wider range to be exploited. However, this solution would imply the addition of a specific antenna on the sensing nodes for Wi-Fi communication, increasing the platform’s complexity and cost.

## 5. Conclusions

In conclusion, the data quality assessment conducted in this study aimed to point out particular network configurations that both reduce hardware conflicts and enhance the quality of the data collected by the platform under analysis. A quantitative performance validation of both wireless engines assessed the viability of the dual-protocol approach in a remote monitoring scenario, suggesting the feasibility of a home monitoring system with wearable sensing nodes and an environmental gateway. This overall evaluation is intended as a robust starting point for further exploration of the technical performance that can be achieved with the proposed solution.

## Figures and Tables

**Table 1:** Outcomes (in terms of statistics and *p*-value) of the Kolmogorov–Smirnov test on the two distributions under analysis in the BLE configurations study.

	Statistic	*p*-Value
with Dynamic Adaptation	0.236	0.001
without Dynamic Adaptation	0.626	9.146 × $10^{-25}$

**Table 2:** Outcomes (in terms of statistic and *p*-values) of the Kolmogorov–Smirnov test on the seven distributions under analysis in the frequency range study.

	Statistic	*p*-Value
10 Hz	0.551	6.524 × $10^{-16}$
20 Hz	0.331	4.650 × $10^{-6}$
30 Hz	0.150	0.161
40 Hz	0.370	3.517 × $10^{-5}$
50 Hz	0.353	1.552 × $10^{-5}$
60 Hz	0.320	7.187 × $10^{-5}$
70 Hz	0.320	2.679 × $10^{-5}$

**Table 3:** Outcomes (in terms of *p*-values) of Dunn’s test for the pairwise analysis of the seven distributions in the frequency range study.

	10 Hz	20 Hz	30 Hz	40 Hz	50 Hz	60 Hz	70 Hz
10 Hz	1.000	0.003	0.008	0.017	0.432	0.877	0.166
20 Hz	0.003	1.000	0.758	0.755	0.040	0.006	0.115
30 Hz	0.008	0.758	1.000	0.974	0.081	0.016	0.210
40 Hz	0.017	0.755	0.974	1.000	0.117	0.029	0.268
50 Hz	0.432	0.040	0.081	0.117	1.000	0.538	0.590
60 Hz	0.877	0.006	0.016	0.029	0.538	1.000	0.234
70 Hz	0.166	0.115	0.210	0.268	0.590	0.234	1.000

**Table 4:** Flushing time required for each step across the different tested scenarios, showing the values of the mean and standard deviation. The value of the Efficiency Index is computed by dividing the sampling time (10 s) by the mean value of the total acquisition time for each scenario.

	Wearable	Environmental: Same Room	Environmental: 1 Room Apart	Environmental: 2 Rooms Apart	Environmental: 3 Rooms Apart
SN to MN [s]	9.65 ± 0.21	9.93 ± 0.30	9.94 ± 0.41	9.74 ± 0.19	9.82 ± 0.22
MN to GATEWAY (MN data) [s]	4.23 ± 0.15	4.49 ± 0.15	4.72 ± 0.27	9.86 ± 1.48	15.27 ± 1.77
MN to GATEWAY (SN data) [s]	4.36 ± 0.12	4.63 ± 0.17	4.83 ± 0.29	10.00 ± 1.32	15.75 ± 1.51
Efficiency Index [dimensionless]	0.36	0.35	0.34	0.25	0.19

## Data Availability

Collected data are available at Kalpa S.r.l.’s data repository.

## Abbreviations

The following abbreviations are used in this manuscript:

BLE	Bluetooth Low Energy
ADC	Analog-to-Digital Converter
ECG	Electrocardiogram
PPG	Photoplethysmogram
m	Meter (International System of Units)
cm	Centimeter (International System of Units)
s	Second (International System of Units)

## References

[1] Demidov A.V., Papshev D.V., Krivonogov L.Y. Principles of Construction, Structure and Features of the ECG and Blood Pressure Monitoring System. Proceedings of the 2020 Moscow Workshop on Electronic and Networking Technologies (MWENT).

[2] Palumbo A., Vizza P., Calabrese B., Ielpo N. (2021). Biopotential Signal Monitoring Systems in Rehabilitation: A Review. Sensors.

[3] Brown L., van de Molengraft J., Yazicioglu R.F., Torfs T., Penders J., Van Hoof C. A low-power, wireless, 8-channel EEG monitoring headset. Proceedings of the 2010 Annual International Conference of the IEEE Engineering in Medicine and Biology.

[4] Zhang Y., Zhou C., Zhongyi H., Xuesong Y. Development of a Continuous Blood Pressure Monitoring System based on Pulse Transit Time and Hemodynamic Covariates. Proceedings of the 13th International Conference on Biomedical Electronics and Devices.

[5] Serrani A., Aliverti A. Feasibility study of an embedded platform for biopotentials acquisition during sports activities. Proceedings of the 2022 IEEE International Workshop on Sport, Technology and Research (STAR).

[6] Serrani A., Aliverti A. Data Quality Assessment for the Validation of Synchronization Performance in an Innovative Wireless Multi-Node Monitoring Platform. Proceedings of the 2023 IEEE International Conference on Metrology for eXtended Reality, Artificial Intelligence and Neural Engineering (MetroXRAINE).

[7] nRF52840 Dongle—Documentation. https://www.nordicsemi.com/Products/nRF52840.

[8] Bluetooth Low Energy Protocol Stack—Documentation. https://infocenter.nordicsemi.com/topic/sds_s140/SDS/s1xx/ble_protocol_stack/ble_protocol_stack.html.

[9] Römer K., Blum P., Meier L., Stojmenović I. (2005). Time Synchronization and Calibration in Wireless Sensor Networks. Handbook of Sensor Networks: Algorithms and Architectures.

[10] Mock M., Frings R., Nett E., Trikaliotis S. Clock synchronization for wireless local area networks. Proceedings of the 12th Euromicro Conference on Real-Time Systems (Euromicro RTS 2000).

[11] Dian F.J., Yousefi A., Somaratne K. Performance evaluation of time synchronization using current consumption pattern of BLE devices. Proceedings of the 2018 IEEE 8th Annual Computing and Communication Workshop and Conference (CCWC).

[12] Tosi J., Taffoni F., Santacatterina M., Sannino R., Formica D. (2017). Performance Evaluation of Bluetooth Low Energy: A Systematic Review. Sensors.

[13] Gomez C., Oller J., Paradells J. (2012). Overview and Evaluation of Bluetooth Low Energy: An Emerging Low-Power Wireless Technology. Sensors.

[14] Liu J., Chen C., Ma Y., Xu Y. Energy Analysis of Device Discovery for Bluetooth Low Energy. Proceedings of the 2013 IEEE 78th Vehicular Technology Conference (VTC Fall).

[15] Buckley J., Aherne K., O’Flynn B., Barton J., Murphy A., O’Mathuna C. Antenna performance measurements using wireless sensor networks. Proceedings of the 56th Electronic Components and Technology Conference 2006.

[16] NRF52840 Bluetooth® 5 Evaluation Board: Datasheet, Nrf52832 VS Nrf52840, and Applications. https://www.utmel.com/components/nrf52840-bluetooth-5-evaluation-board-video-datasheet-nrf52832-vs-nrf52840-and-applications?id=1823.

[17] Bluetooth® Low Energy Connections. https://developerhelp.microchip.com/xwiki/bin/view/applications/ble/introduction/bluetooth-architecture/bluetooth-controller-layer/bluetooth-link-layer/Connections.

[18] Generic Access Profile (GAP). https://software-dl.ti.com/lprf/simplelink_cc2640r2_sdk/1.35.00.33/exports/docs/ble5stack/ble_user_guide/html/ble-stack/gap.html.

